# Thermo-Hydrodynamic Effect of Gas Split Floating Ring Seal with Rayleigh Step Grooves

**DOI:** 10.3390/ma16062283

**Published:** 2023-03-12

**Authors:** Shaoxian Bai, Dongdong Chu, Chunhong Ma, Jing Yang, Shiyi Bao

**Affiliations:** 1 College of Mechanical Engineering, Zhejiang University of Technology, Hangzhou 310032, China; 2 School of Mechanical and Energy Engineering, Zhejiang University of Science and Technology, Hangzhou 310023, China

**Keywords:** thermo-hydrodynamic effect, gas lubrication, split floating ring seal, Rayleigh step groove, deflection angle

## Abstract

The force equilibrium and moment equilibrium play a significant role on the sealing performance of gas split floating ring seals. A small deflection angle may generate seriously wear on sealing surface and cause seal failure. Therefore, the thermo-hydrodynamic lubrication analysis of gas split floating ring seal with Rayleigh grooves is investigated considering the deflection angle and frictional heat of surface contact, which is beneficial to grasp the hydrodynamic characteristics and rules under high-temperature and high-speed conditions. Pressure and temperature distributions of sealing rings are numerically calculated for the cases with different deflection angle, rational speed, seal pressure and ambient temperature. Then, the hydrodynamic effect and sealing performance are analyzed. The obtained results show that, the surface Rayleigh step grooves do not present obvious hydrodynamic effect when split seal ring has no deflection. While, a significant hydrodynamic effect can be obtained when the split seal ring presents a deflection angle about dozens of micro radians. Here, a 10% increase of opening force is achieved when the deflection angle reaches 80 μrad in the case of speed 30,000 r/min and seal pressure 0.2 MPa. Moreover, the hydrodynamic effect becomes obvious with increasing deflection angle as well as rotational speed. Meanwhile, the growth of rotational speed results in an obvious increase of film temperature. The increase of ambient temperature has a significant influence on the decrease of leakage rate. When the ambient temperature increases from 340 K to 540 K, the leakage rate reduces exceeding 50%, however, it does not present obvious effect on the opening force. The proposed model has the potential to provide the theoretical basis and design guidance for surface grooves of gas split floating ring seal in the future.

## 1. Introduction

With the rapid promotion of rotational speed and working temperature, split floating ring seal has been widely used in gas turbine engine, liquid-propellant rocket engine and other high parameter equipment. Hydrodynamic effect plays an important role for seals to keep non-contact running and reduce contact wear so as to obtain a long service life. Rayleigh step groove, as a typical groove, has been widely used in the design of gas split floating ring seals in order to improve its hydrodynamic opening force [1,2,3,4]. However, under the variable working conditions of engine and other power units with multiple speeds and varying temperatures, the thermo-viscosity effect will further lead to the variation of hydrodynamic effect and affect the stability of sealing operation [5,6,7,8,9].

Back in the 1970s and 1980s, it was found that Rayleigh step groove on the surface of graphite seal ring can effectively reduce the leakage rate and seal wear [10,11,12,13]. For example, Hady and Ludwig’s experimental work [14] proved that adding Rayleigh step groove on the surface of circumferential seal ring could significantly reduce the friction torque and surface temperature at room temperature in 1972. Afterwards, the study of Oike [15] and Kikuchi [16] showed that the opening force is obviously related to the depth of Rayleigh step groove. Tran and Haselbacher’s analysis on Rayleigh step groove [17] presented that, the overall leakage of radial seal in the form of split values was only 7% of that of ordinary seal. Recently, Arghir [18] and Dahit [19,20] established a numerical calculation model considering unsteady Reynolds equation and film discontinuity. Their analysis showed that, for large compression coefficient, the hydrodynamic opening force generated by surface grooves could increase more than 25%. Besides, their thermodynamic analysis illustrated that, the excessive groove depth of Rayleigh step groove was not conducive to the opening of seal. The couple of groove depth and large pressure difference would lead to a decrease of seal film thickness. Meanwhile, numerical analysis by Ding [21] show that, the convergent clearance formed by the deflection of seal ring could produce significant hydrodynamic effect, which could make the equilibrium film thickness increase from about 1.5 μm to 7.0μm with rotational speed increasing form 10,000 r/min to 100,000 r/min, for the isothermal condition with seal pressure 0.4 MPa. 

The hydrodynamic effect is the key factor to determine the sealing performance and service life of gas split floating ring seals. However, the related work conducted on these seals is mainly focused on the isothermal condition currently. In field applications, the high temperature may aggravate the seal wear obviously, which can cause the failure of seals finally. Therefore, the novelty of this study is coupling the temperature field to investigate the thermo-hydrodynamic behaviors of gas split floating ring seal with Rayleigh step grooves. The influence of deflection angle, rotational speed, seal pressure and ambient temperature on the thermo-hydrodynamic effect and the temperature rise is calculated numerically, which can provide design reference for enhancing the hydrodynamic opening force and reducing the seal surface temperature rise.

## 2. Theoretical Model

### 2.1. Geometric Model

**Figure 1:**
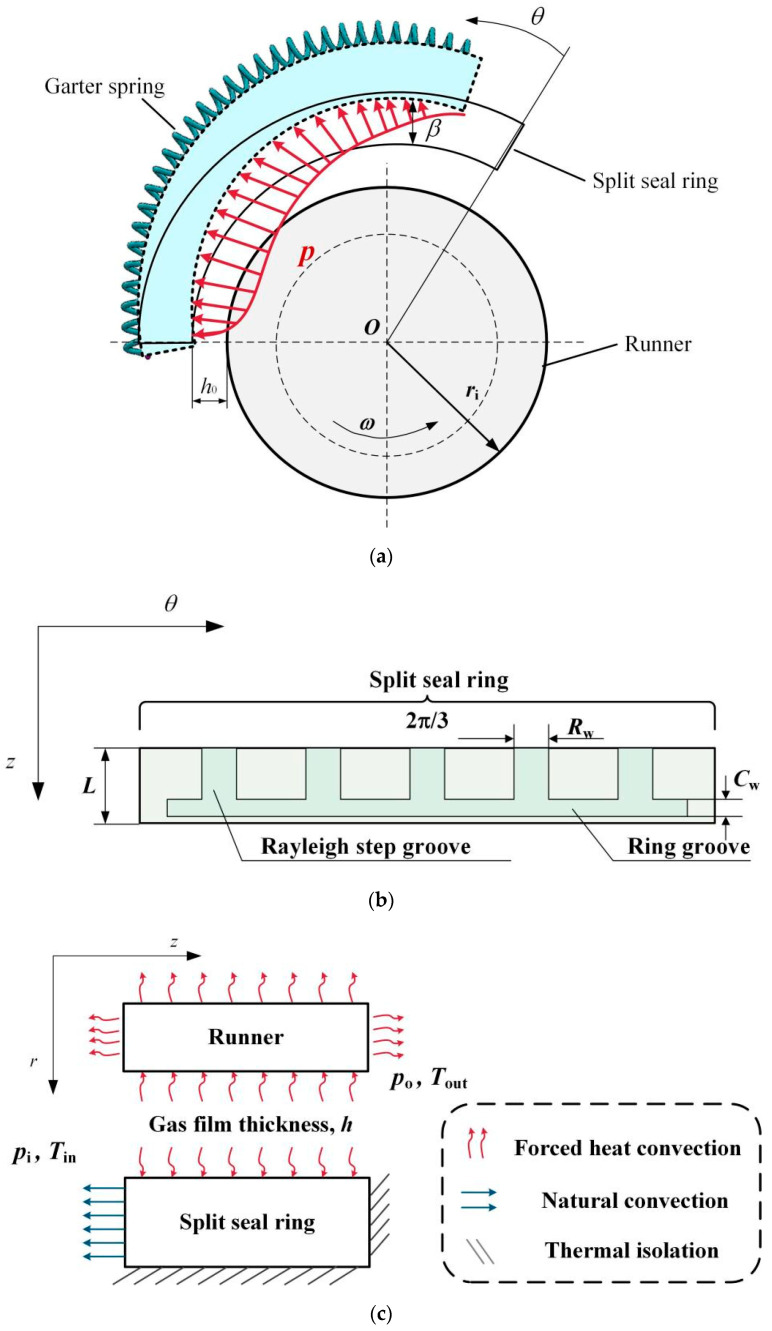
Schematic diagram of split floating ring seal structure and heat transfer boundary [2]. (**a**) Split floating ring seal structure; (**b**) Schematic diagram of Rayleigh step groove structure; (**c**) Boundary conditions.

The schematic diagram of the split floating ring seal and thermal boundary conditions is shown in Figure 1. In the static state, the split seal ring is assembled into a whole by the circumferential spring, and holds the runner under the action of circumferential spring force and radial closing force of the high-pressure medium cavity, forming a contact seal; when the runner is rotating, the runner and the seal ring are actually in a full-film or mixed lubrication state due to changes in operating parameters such as sealing pressure and rotation speed; solid contact friction not only affects the temperature distribution of the seal interface but also leads to wear.

The three-split seal ring structure is selected, and any one of the split seal rings can be used as an example for analysis. The split seal ring surface is designed with Rayleigh step grooves and circumferential groove, as shown in Figure 1b.

For the split ring surface, the local film thickness [22], *h*(*r*,*θ*), is given by

(1) $$h\left(r,\theta \right)=\left\{\begin{array}{c} \begin{array}{cc} h_{0}+h_{d}+\left(h_{0}+r_{i}\right)\beta \left(\frac{2\pi }{3}-\theta \right), & \begin{array}{cc} \mathrm{groove} & \mathrm{area} \end{array} \end{array}\\ \begin{array}{cc} h_{0}+\left(h_{0}+r_{i}\right)\beta \left(\frac{2\pi }{3}-\theta \right), & \begin{array}{cc} \;\;\mathrm{Non-groove} & \mathrm{area} \end{array} \end{array} \end{array}\right.$$

where *h*_0_ is the gas film base thickness, m; *h*_d_ is the Rayleigh step groove depth, m; *β* is the split ring deflection angle, rad; *θ* is the attitude angle, rad; and *r*_i_ is the inner diameter of seal ring, m. 

The specific structure parameters of the seal are shown in Table 1.

The materials of the moving ring and the static ring are stainless steel and graphite, respectively. The material parameters for calculation are shown in Table 2.

### 2.2. Theoretical Model

Fluid lubrication based on the Reynolds equation has been widely applied in the numerical analysis of seals. For gas thermos-hydrodynamic lubrication analysis of seals, the mathematical model mainly includes the Reynolds equation, energy equation, solid heat conduction equation and gas state equations [22].

Considering the effect of surface roughness, Reynolds equation for sealing gas of split floating ring seal with the introduction of the PC average flow model [23] and the contact factor proposed by Wu et al. [24] is:

(2) $$\frac{\partial }{r_{i}\partial \theta }\left(Q_{\theta }\frac{\rho h^{3}}{\eta }\frac{\partial p}{r_{i}\partial \theta }\right)+\frac{\partial }{\partial z}\left(Q_{z}\frac{\rho h^{3}r}{\eta }\frac{\partial p}{\partial z}\right)=6\omega Q_{s}\frac{\partial \left(\rho h\right)}{\partial \theta }$$

where *h* is the thickness of gas film, m; *p* is the gas film pressure, Pa; *ρ* is the density, kg∙m^−3^; *θ* is the rotation angle, rad; *z* is the dimension along the film thickness direction, m; *Q*_θ_ is the circumferential pressure flow factor; *Q*_z_ is the axial pressure flow factor; *Q*_s_ is the shear flow factor; *η* is the viscosity, Pa∙s; and *ω* is the rotational speed, r/min.

Considering the heat of contact friction on the solid surface, the energy equation of the sealed gas film [22] can be modified to the following form [25]:

(3) $$\begin{array}{l} \left(Q_{s}\frac{\omega r_{i}h}{\eta }-Q_{\theta }\frac{h^{3}}{12\eta }\frac{\partial p}{r_{i}\partial \theta }\right)\frac{\partial T}{r_{i}\partial \theta }-\frac{\partial }{\partial z}\left(Q_{z}\frac{h^{3}}{12\eta }\frac{\partial p}{\partial z}\right)\frac{\partial T}{\partial z}\\ =\frac{\eta \omega^{2}r_{i}^{2}}{h\rho c_{v}}-\frac{h^{3}}{12\eta \rho c_{v}}\left[{\left(\frac{\partial p}{r_{i}\partial \theta }\right)}^{2}+{\left(\frac{\partial p}{\partial z}\right)}^{2}\right]+\frac{fp_{c}\omega r_{i}}{\rho c_{v}}\\ +\frac{k_{g,s1}}{\rho c_{v}}\left(T_{s1}-T\right)+\frac{k_{g,s2}}{\rho c_{v}}\left(T_{s2}-T\right) \end{array}$$

where, *p*_c_ is the bearing capacity per unit area of the contact surface rough peak [26]; *T*_s_ is the solid temperature, K; *c*_v_ is the specific heat capacity at constant volume, J/(kg·K); *k*_g,s1_ and *k*_g,s2_ are the thermal convective heat transfer coefficients at the interface of relative motion, W/(m^2^∙K), respectively; *T*_s1_ and *T*_s2_ are the solid surface temperatures at the interface of relative motion, K.

(4) $$p_{c}=\frac{16\sqrt{2}}{15}\pi {\left(\mu \alpha \sigma \right)}^{2}\sqrt{\frac{\sigma }{\alpha }}EF_{2.5}(\lambda )$$

(5) $$\frac{1}{E}=\frac{1}{2}\left(\frac{1-v_{1}^{2}}{E_{1}}+\frac{1-v_{2}^{2}}{E_{2}}\right)$$

The calculation in this paper does not consider the influence of surface waviness for the time being, and assuming that the height of surface roughness peak conforms to the Gaussian distribution law, the formula for *F*_2.5_(*λ*) is

(6) $$F_{2.5}(\lambda )=\frac{1}{\sqrt{2\pi }}\int_{\lambda }^{\infty }{\left(s-\lambda \right)}^{2.5}e^{-\frac{s^{2}}{2}}ds$$

where, *μ*, *α, σ* are roughness characterization parameters, taking the value range of *μασ* from 0.04 to 0.08 [22]. *λ* is the film thickness ratio, *λ* = *h*/*σ. E* is the integrated elastic modulus. *E*_1_ and *E*_2_ are the elastic moduli of the two contact surfaces, N/mm^2^, respectively. *v*_1_ and *v*_2_ are Poisson’s ratios.

The heat transfer equation of the seal ring is

(7) $$\frac{\partial^{2}T_{s}}{r^{2}\partial \theta^{2}}+\frac{1}{r}\frac{\partial }{\partial r}\left(r\frac{\partial T_{s}}{\partial r}\right)+\frac{\partial^{2}T_{s}}{\partial z^{2}}=0$$

The heat transfer equation of the seal runner is

(8) $$\frac{k_{c,2}}{\rho_{s,2}c_{s,2}}\left[\frac{\partial^{2}T_{s}}{r^{2}\partial \theta^{2}}+\frac{1}{r}\frac{\partial }{\partial r}\left(r\frac{\partial T_{s}}{\partial r}\right)+\frac{\partial^{2}T_{s}}{\partial z^{2}}\right]=\omega \frac{T_{s}}{\partial \theta }$$

where *k*_c_ is the heat transfer coefficient of the material, W/(m·K). *ρ* is the density, kg∙m^−3^. *c*_s_ is the specific heat capacity, J/(kg·K).

In this analysis, air is applied as sealing medium. For the above equations, considering the compressibility of real gas, the state equation of pressure, *p*, can be described as

(9) $$p=\varepsilon c_{p}\rho i_{d}E_{m}$$

The gas film temperature equation is expressed as

(10) $$T=\frac{i_{d}E_{m}}{c_{v}}$$

where, *ε* is the compressibility coefficient of the gas, *i*_d_ is the degrees of freedom of motion of gas molecules, *c*_p_ is the pressure constant coefficient (*c*_p_ = *R*_u_/*c*_v_), *R*_u_ is the ideal gas constant, *E*_m_ is the energy of gas molecular per freedom. Here, the database REFPROP published by NIST is used to obtain the air properties.

Based on the actual geometry of the split floating ring seal, forced convective heat transfer, natural convective heat transfer, and adiabatic boundary conditions exist between the split seal ring, runner, sealing gas film, and the environment, as shown in Figure 1c.

The circumferential periodic temperature boundary conditions are

(11) $$T\left(\theta ,z=0\right)=T\left(\theta ,z=\frac{2\pi }{N}\right)$$

where, *N* is the number of split seal ring, and *N* = 3 is taken here.

The axial dynamic temperature boundary conditions are

(12) $$\mathrm{if}\;q_{z}\left(\theta ,z=0\right)<0,\;T\left(\theta ,z=0\right)=T_{\mathrm{in}}$$

(13) $$\mathrm{if}\;q_{z}\left(\theta ,z=L\right)>0,\;T\left(\theta ,z=L\right)=T_{\mathrm{out}}$$

where,

(14) $$q_{z}=-\frac{r_{i}h^{3}}{12\eta }\frac{\partial p}{\partial z}$$

The average pressure of the sealed gas film is

(15) $$p_{\mathrm{av}}=\frac{\int_{0}^{\frac{2\pi }{3}}\int_{0}^{L}pr_{i}d\theta dz}{\int_{0}^{\frac{2\pi }{3}}\int_{0}^{L}r_{i}d\theta dz}$$

The leakage rate of the seal is

(16) $$Q=\int_{0}^{\frac{2\pi }{3}}\frac{p}{p_{a}}\left(-\frac{r_{i}h^{3}}{12\eta }\frac{\partial p}{\partial z}\right)d\theta$$

In order to analyze the performance of hydrodynamic effect, two rate parameters are defined. One is the rate of increase in the average pressure, *R*_P_, which reflects the increase of opening force under shear, the expression is

(17) $$R_{p}=\frac{p_{\mathrm{av}}-p_{\mathrm{av},\omega =0}}{p_{\mathrm{av},\omega =0}}$$

The other parameter is the increase rate of leakage rate, which reflects the increase of leakage rate under shear, *R*_Q_, and its expression is

(18) $$R_{Q}=\frac{Q-Q_{\omega =0}}{Q_{\omega =0}}$$

In this numerical study, as discussed in our pervious work [22], the finite difference method was used to solve the governing equations to obtain the pressure distribution, the temperature distribution and sealing performance. The simulation codes in this work were written by authors.

### 2.3. Model Validation

**Figure 2:**
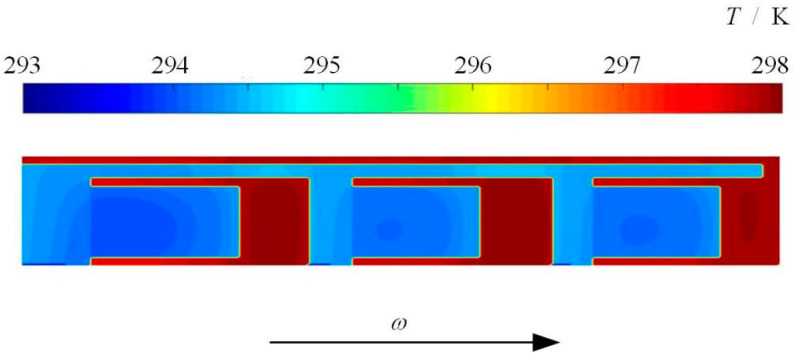
Film temperature distribution obtained by present model for the reference split floating ring seal with pocket depth 10 μm [20]. (*p*_i_ = 0.1 MPa, *p*_o_ = 0.6 MPa, *ω* = 1000 s^−1^, *T*_in_ = 293 K, *T*_out_ = 293 K).

In order to validate the model, the temperature rise between the referenced work of Dahite [20] and this proposed model were compared. In Dahite’s numerical analysis, it was shown that the heating is greater on the first pad according to the direction of rotation, which is consistent with the fact that the minimum film thickness is at the leading edge of the split ring. As shown in Figure 2, the same film temperature distribution can also be obtained by the present model. That is the low film thickness often result in high temperature rise. 

**Figure 3:**
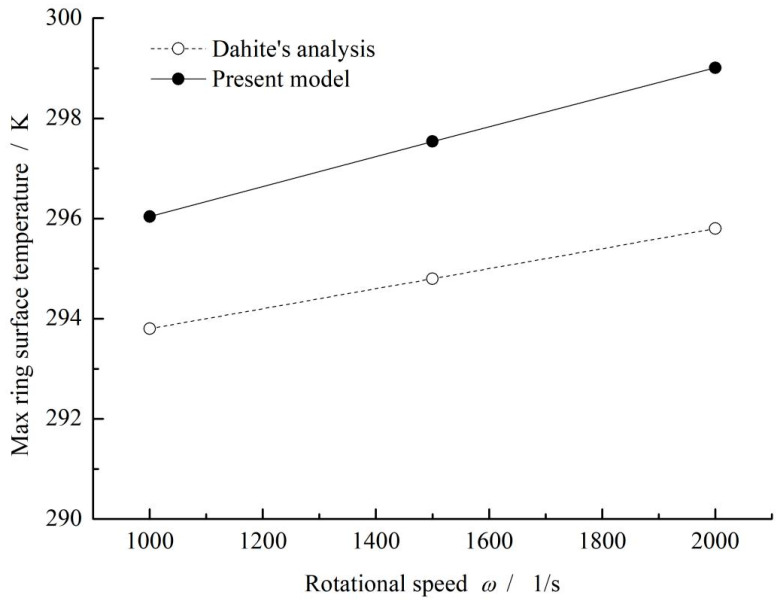
Influence of rotational speed on max ring surface temperature. (*p*_i_ = 0.1 MPa, *p*_o_ = 0.6 MPa, *ω* = 1000 s^−1^, *T*_in_ = 293 K, *T*_out_ = 293 K).

Further, the max ring surface temperature calculated by the present model is also compared with the reference, as shown in Figure 3. It can be seen that the max ring surface temperature increases from about 296 K to 299 K with increasing rotational speed from 1000 s^−1^ to 2000 s^−1^. As a whole, the theoretical results of the present model are in good agreement with Dahite’s work. 

## 3. Results and Discussion

**Figure 4:**
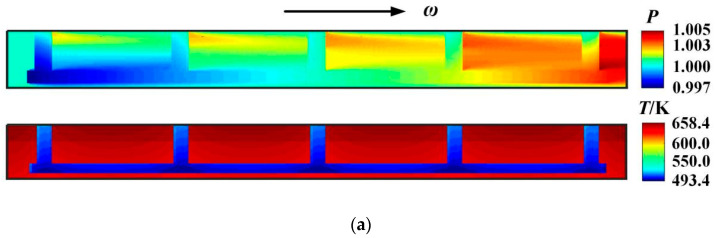

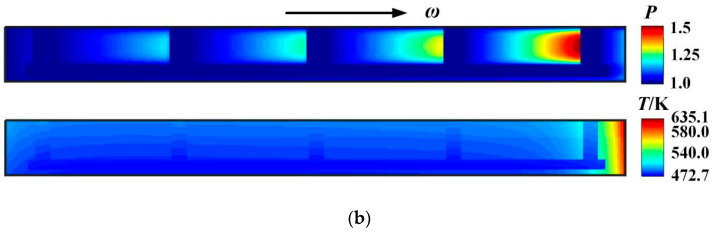
Pressure distribution and temperature distribution of split floating ring seal. (**a**) *β* = 0 rad; (**b**) *β* = 20 × 10^−6^ rad (*p*_i_ = 0.1 MPa, *ω* = 3.0 × 10^4^ r/min, *T*_in_ = 580 K, *T*_out_ = 380 K, *β* = 0/2e^−5^ rad).

Figure 4 shows the pressure and temperature distribution of the split floating ring seal film. It can be seen that the deflection angle and surface groove have an obvious influence on the pressure distribution and temperature. For the sealing pressure distribution, under the working conditions of seal pressure *p*_i_ = 0.1 MPa and rotational speed *ω* = 3.0 × 10^4^ r/min, the ring deflection forms a wedge convergence gap, which causes significant dynamic pressure effect along the direction of rotational rotation. When the deflection angle *β* increases from 0 rad to 20 × 10^−6^ rad, the maximum film pressure increases from 0.1005 MPa to 0.15 MPa, while the maximum film temperature decreases from 658.4 K to 635.1 K.

**Figure 5:**
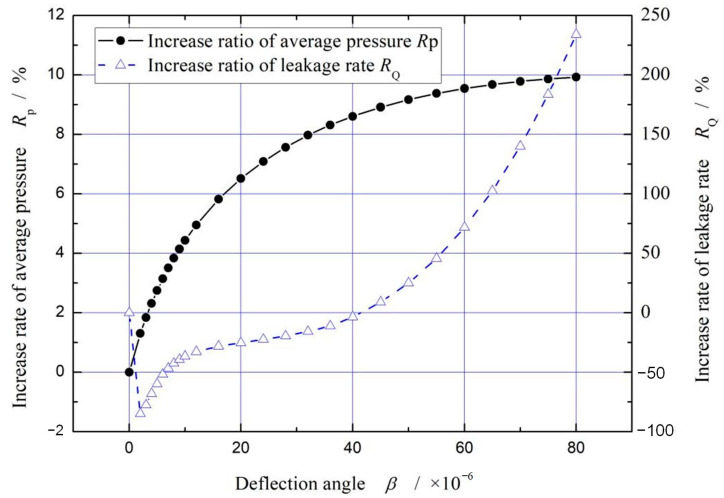
Influence of deflection angle on dimensionless mean pressure and leakage. (*p*_i_ = 0.2 MPa, *ω* = 3.0 × 10^4^ r/min, *T*_in_ = 580 K, *T*_out_ = 380 K, *p*_s_ = 0.03 MPa).

Figure 5 illustrates the influence of deflection angles on increase rate of average pressure and increase rate of leakage rate. As shown in this figure, when the deflection angle increases from 0 to 80 × 10^−6^ rad, increase rate of average pressure increases rapidly, from 0 to about 10%. As a whole, the increase rate of leakage increases continuously with the increase of deflection angle, reaching a maximum value about 240%, except for decrease not exceeding about 84.8% when *β* < 4.5 × 10^−6^ rad.

**Figure 6:**
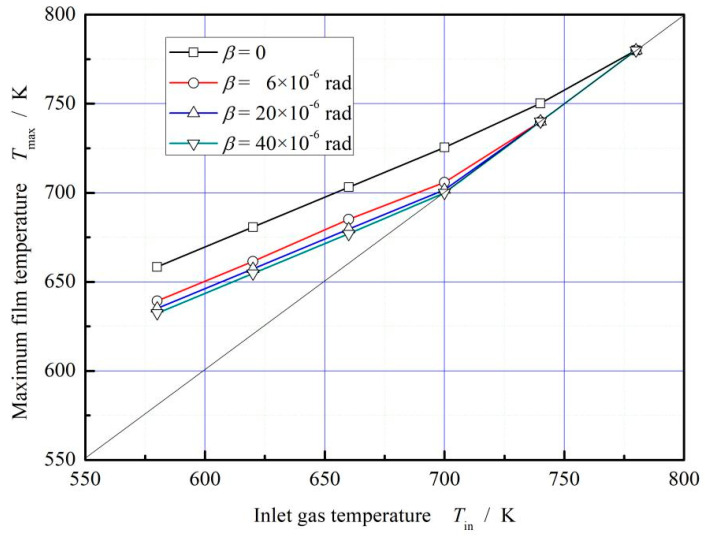
Influence of deflection angle on the maximum film temperature. (*p*_i_ = 0.2 MPa, *ω* = 3.0 × 10^4^ r/min, *T*_out_ = 380 K).

Figure 6 presents the influence of deflection angle on maximum film temperature under different sealing temperature. It is shown that, the maximum film temperature increases with increasing sealing temperature. The more important is that, the maximum film temperature decreases with increasing deflection angle due to the decrease of shear rate, reaching about 3.9% in degree. In next section, the influence of the working parameters hydrodynamic effect, including rotational speed, seal pressure and ambient temperature, will be further discussed.

### 3.1. Rotation Speed

**Figure 7:**
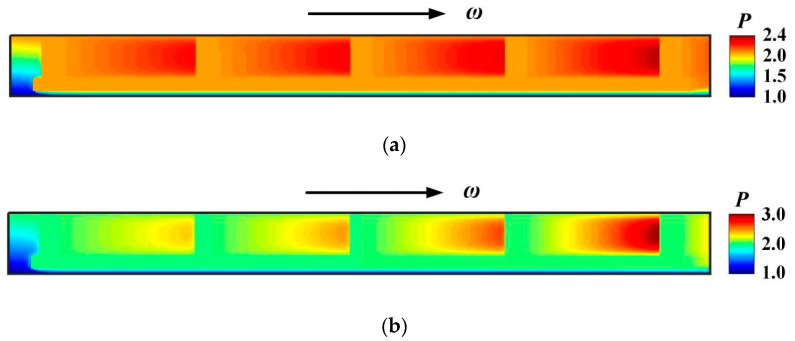
Pressure distribution at different deflection angles. (*p*_i_ = 0.2 MPa, *ω* = 3.2 × 10^4^ r/min, *T*_in_ = 580 K, *T*_out_ = 380 K). (**a**) *β* = 6 × 10^−6^ rad; (**b**) *β* = 20 × 10^−6^ rad.

Figure 7 demonstrates the film pressure distributions under different deflection angles. For the case of seal pressure *p*_i_ = 0.2 MPa and rotational speed *ω* = 3.2 × 10^4^ r/min, compared with the film pressure without deflection, the deflection angle *β* = 60 × 10^−6^ rad may produces obvious hydrodynamic pressure, the maximum film pressure increasing from 2.4 to 3.0.

**Figure 8:**
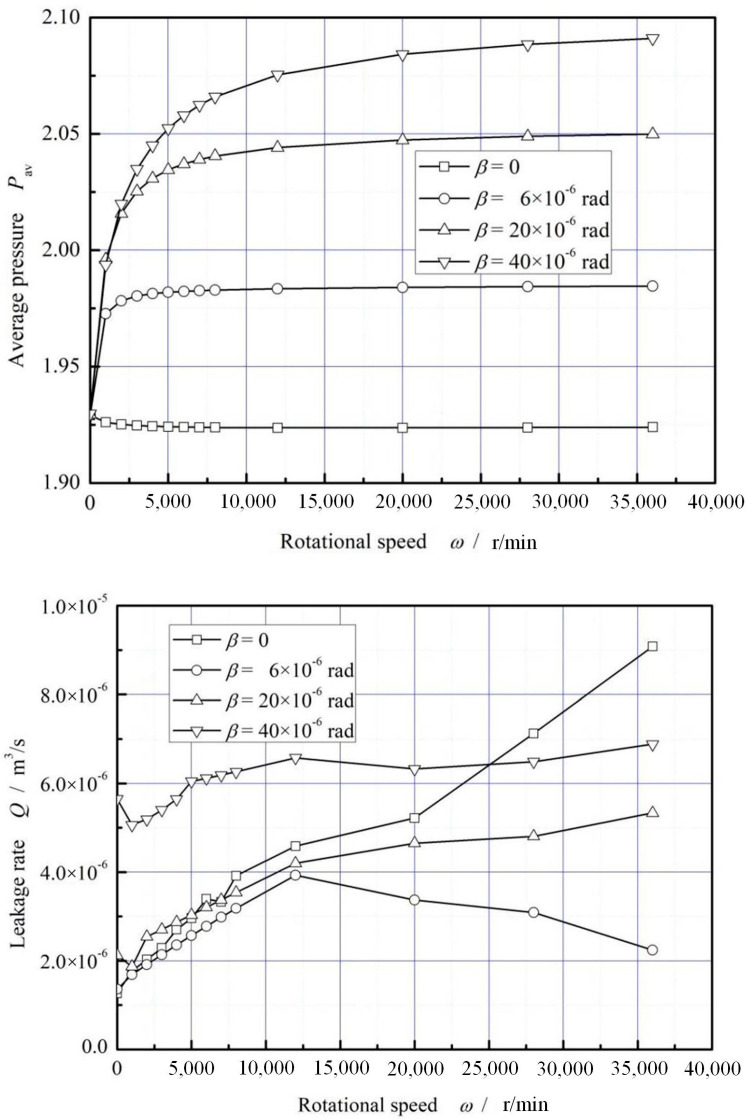
Influence of rotating speed on dimensionless mean pressure and leakage. (*p*_i_ = 0.2 MPa, *T*_in_ = 580 K, *T*_out_ = 380 K).

Figure 8 shows the influence of rotational speed on film average pressure and leakage rate. As can be seen that, the average pressure increases with the increasing rotational speed, but the increase slows down after the rotational speed exceeding 20,000 r/min. Compared with the static condition, the increase of the average pressure may reach about 8.4% in the case of the rotational speed *ω* = 3.6 × 10^4^ r/min and the deflection angle *β* = 20 × 10^−6^ rad. 

In addition, at a small deflection angle *β* = 6 × 10^−6^ rad, the leakage rate increases first and then decreases with the increasing rotational speed. The reason may be that, there is a slight dynamic pressure effect when the seal works at high speed, which prevents the leakage to some certain extent. With the deflection angle continues to increase, the leakage rate also increases with increasing rotational speed.

**Figure 9:**
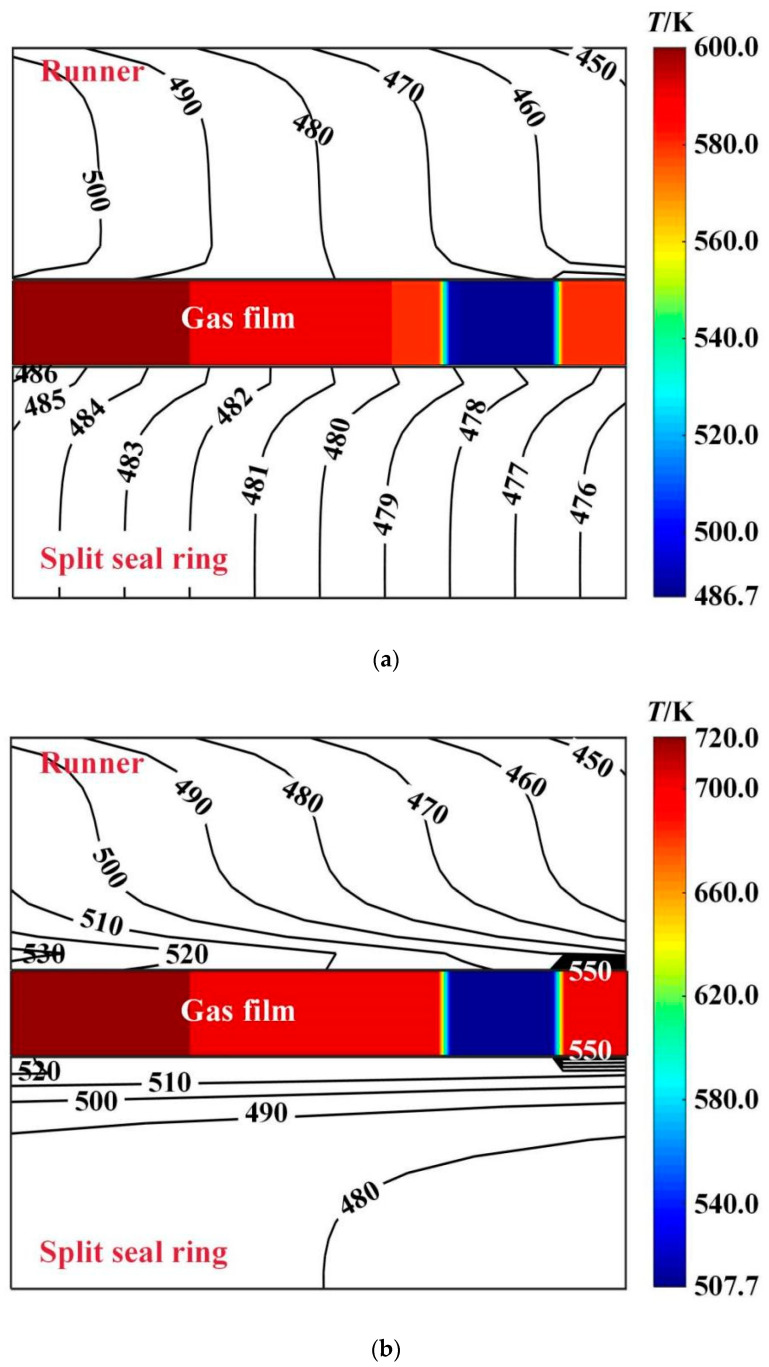
Influence of rotation speed on the maximum temperature of gas film. (*p*_i_ = 0.2 MPa, *ω* = 4.0 × 10^4^ r/min, *T*_in_ = 580 K, *T*_out_ = 380 K). (**a**) *β* = 6 × 10^−6^ rad; (**b**) *β* = 0.

Figure 9 shows the temperature distribution of the center profile of seal friction pair at different deflection angles. As can be seen from this figure, when the deflection angles are 0 and 6 × 10^−6^ rad, respectively, the temperature distributions are quite different. The temperature of the gas film rises rapidly after flowing through the groove area. The maximum temperature of gas film decreased from 720.0 K to 600.0 K, with a decrease of about 16.7%. The runner temperature decreases from 550.0 K to 500.0 K, a decrease of about 9.1%, while the seal ring temperature is reduced from 550.0 K to 486.0 K, a decrease of about 11.6%.

**Figure 10:**
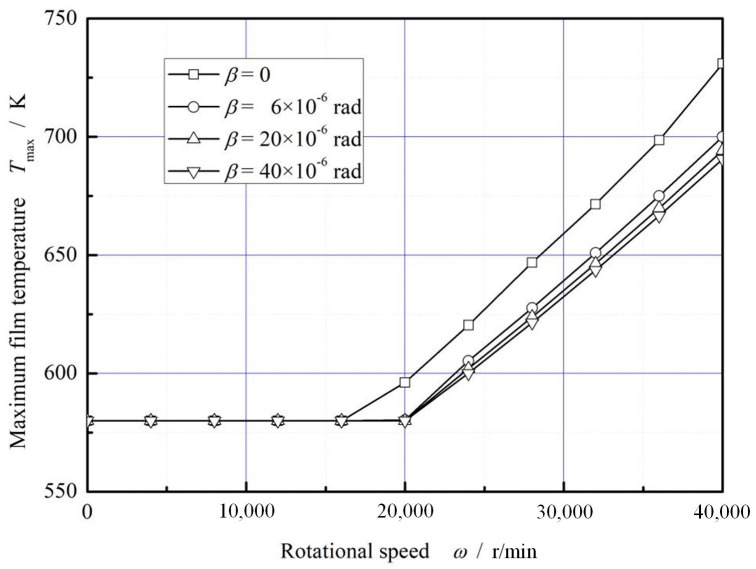
Influence of rotation speed on the maximum temperature of gas film. (*p*_i_ = 0.2 MPa, *T*_in_ = 580 K, *T*_out_ = 380 K).

Figure 10 plots the influence of rotational speed on the maximum film temperature. With the increase of rotational speed, the maximum film temperature increases obviously at high speed. Compared with the case of no deflection, the increase of deflection angle leads to an obvious decrease of the maximum film temperature, about 6.0% in degree.

### 3.2. Seal Pressure

**Figure 11:**
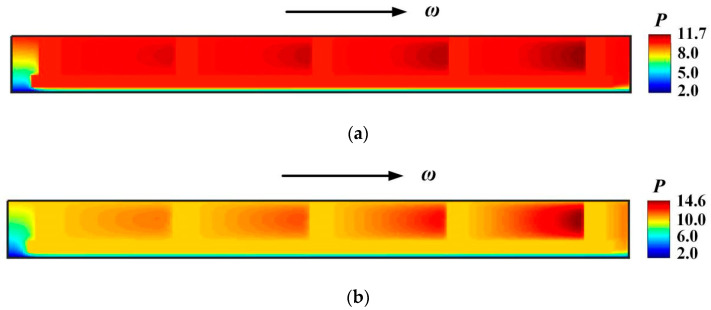
Pressure distribution at different deflection angles. (*p*_i_ = 1.0 MPa, *ω* = 3.0 × 10^4^ r/min, *T*_in_ = 580 K, *T*_out_ = 380 K). (**a**) *β* = 6 × 10^−6^ rad; (**b**) *β* = 20 × 10^−6^ rad.

Figure 11 shows the film pressure distribution under different deflection angles in the case of seal pressure *p*_i_ = 1.0 MPa and *ω* = 3.0 × 10^4^ r/min. It can be seen that the increase of deflection angle produces an obvious hydrodynamic pressure effect at higher seal pressure. 

**Figure 12:**
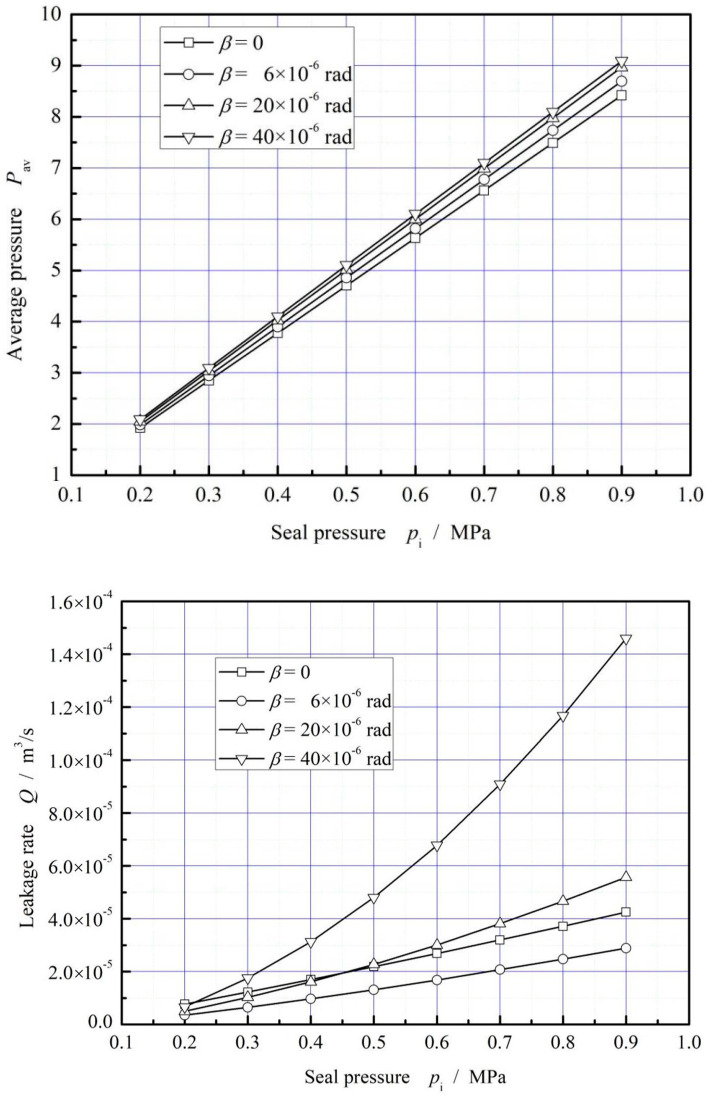
Influence of seal pressure on dimensionless mean pressure and leakage. (*ω* = 3.0 × 10^4^ r/min, *T*_in_ = 580 K, *T*_out_ = 380 K).

Figure 12 illustrates the effect of seal pressure on average film pressure and leakage rate. It is shown that both the average pressure and the leakage rate increase rapidly with the increase of seal pressure. Compared with the influence of the rotational speed on the average pressure, the influence of seal pressure is smaller.

**Figure 13:**
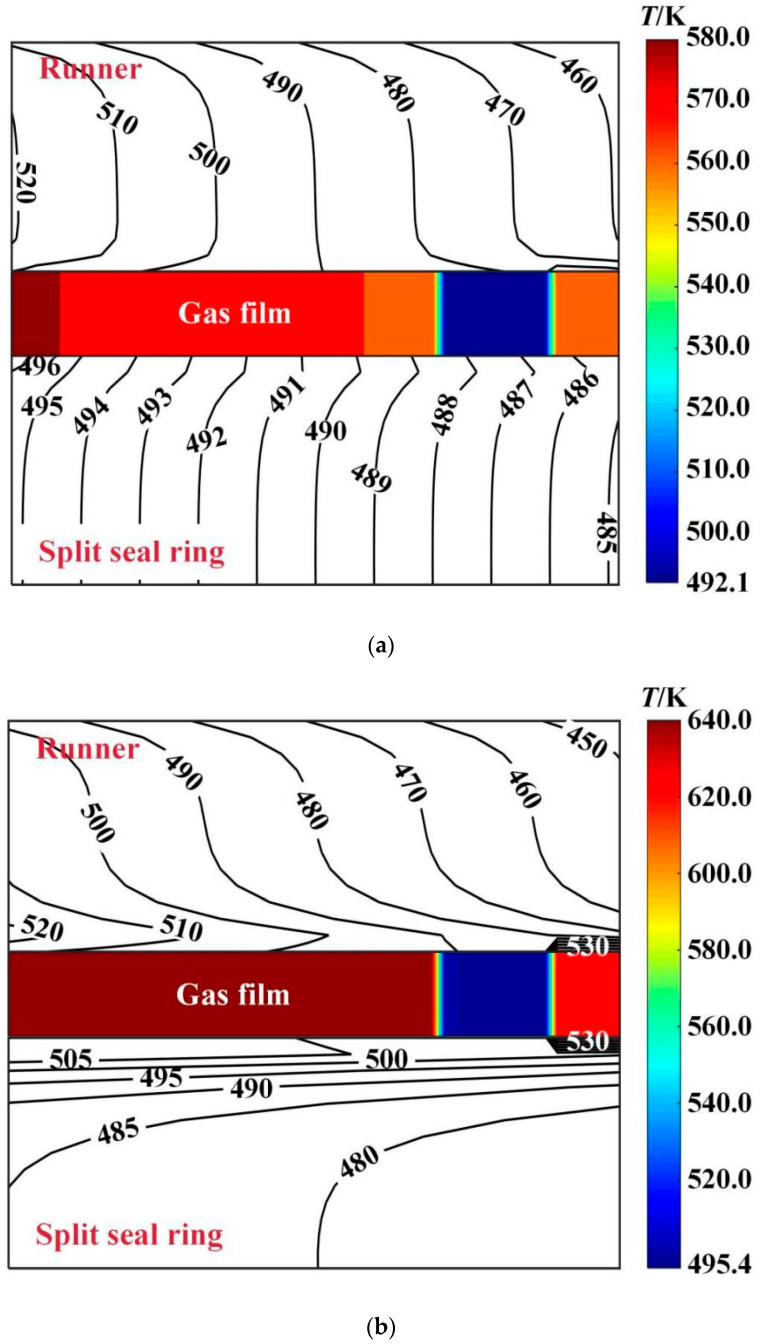
Influence of rotation speed on the maximum temperature of gas film. (**a**) *β* = 6 × 10^−6^ rad; (**b**) *β* = 0; (*p*_i_ = 0.2 MPa, *ω* = 3.0 × 10^4^ r/min, *T*_in_ = 580 K, *T*_out_ = 380 K).

Figure 13 shows the temperature distribution of the center profile of sealing friction pair at different deflection angles. It is shown that the gas film temperature decreases along the high-pressure side and low-pressure side due to the gas expansion and heat absorption. When the deflection angle increases from 0 to 60 × 10^−6^ rad, the maximum film temperature decreases from 640.0 K to 580.0 K, with a decrease of about 12.5%. The runner temperature change is not obvious, while the seal ring temperature is reduced from 530.0 K to 496.0 K, a decrease of about 6.4%.

**Figure 14:**
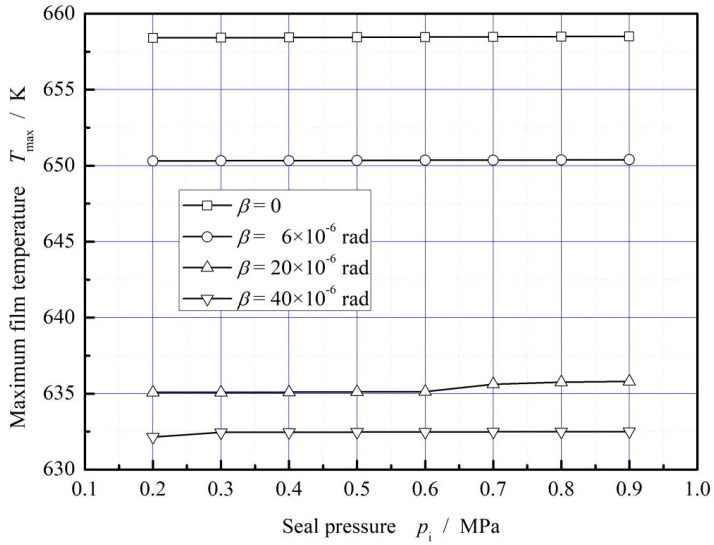
Influence of seal pressure on the maximum temperature of gas film. (*ω* = 3.0 × 10^4^ r/min, *T*_in_ = 580 K, *T*_out_ = 380 K).

Figure 14 plots the influence of seal pressure on maximum film temperature. With the increase of the seal pressure, the maximum film temperature presents no obvious variation. Compared with the absence of ring deflection, the increase of ring deflection angle has a significant decreasing trend on the maximum temperature of the film, and the maximum cooling range is about 4.3%.

### 3.3. Ambient Temperature

**Figure 15:**
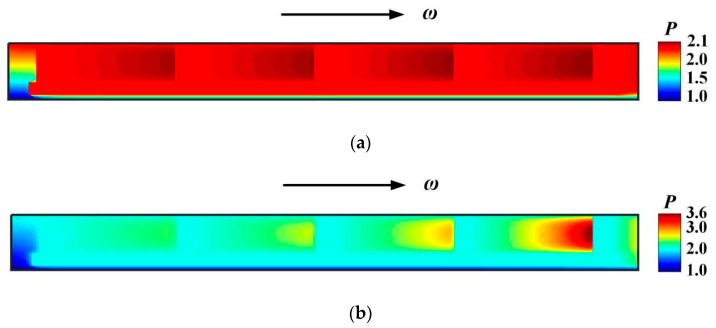
Pressure distribution at different deflection angles. (*p*_i_ = 0.2 MPa, *ω* = 3.0 × 10^4^ r/min, *T*_in_ = 580 K, *T*_out_ = 420 K); (**a**) *β* =2 × 10^−6^ rad; (**b**) *β* = 40 × 10^−6^ rad.

Figure 15 shows the film pressure distribution under a higher ambient temperature *T*_out_ = 420 K. It can be seen that, with the increase of deflection angle, the dynamic pressure effect is also enhanced significantly. The maximum film pressure increases from 2.1 to 3.6 when the deflection angle increases from 2 × 10^−6^ rad to 40 × 10^−6^ rad.

**Figure 16:**
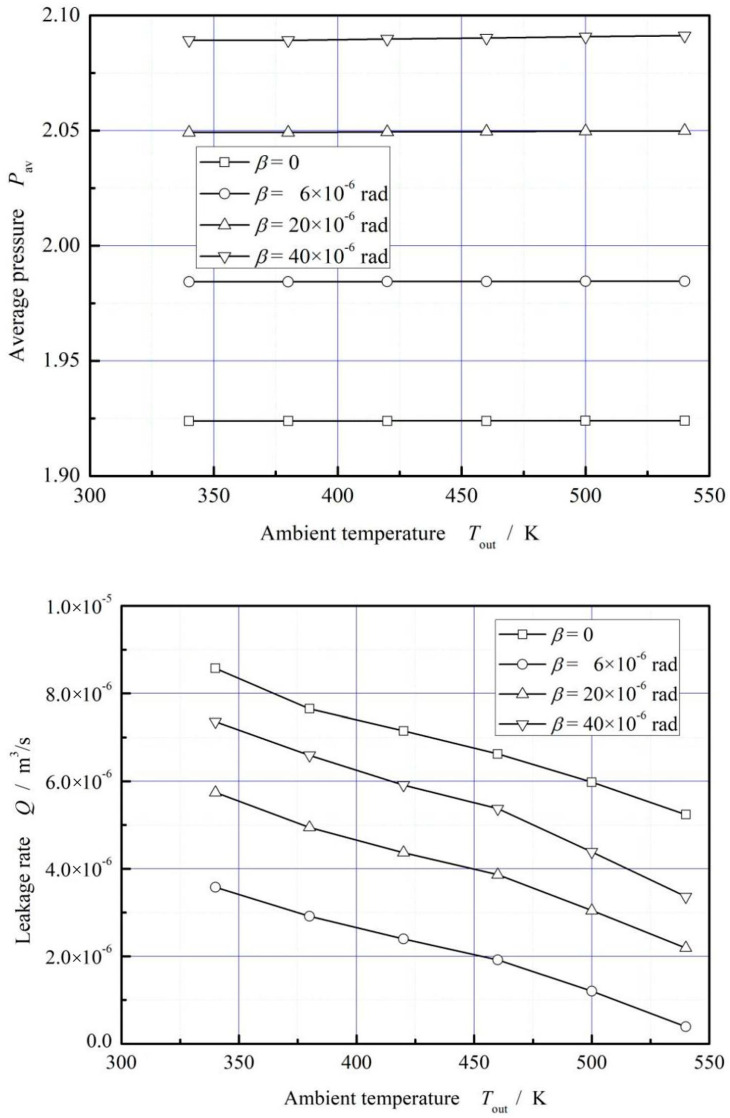
Influence of ambient temperature on dimensionless mean pressure and leakage. (*p*_i_ = 0.2 MPa, *ω* = 3.0 × 10^4^ r/min, *T*_in_ = 580 K).

Figure 16 illustrates the influence of seal pressure on average pressure and leakage rate. It is shown that, the average pressure does not increase significantly with the increase of ambient temperature, but the leakage rate decreases significantly with increasing ambient temperature due to the gas thermo-viscosity effect. The decrease of the leakage rate exceeds 50% when the ambient temperature increases form 340 K to 540 K.

**Figure 17:**
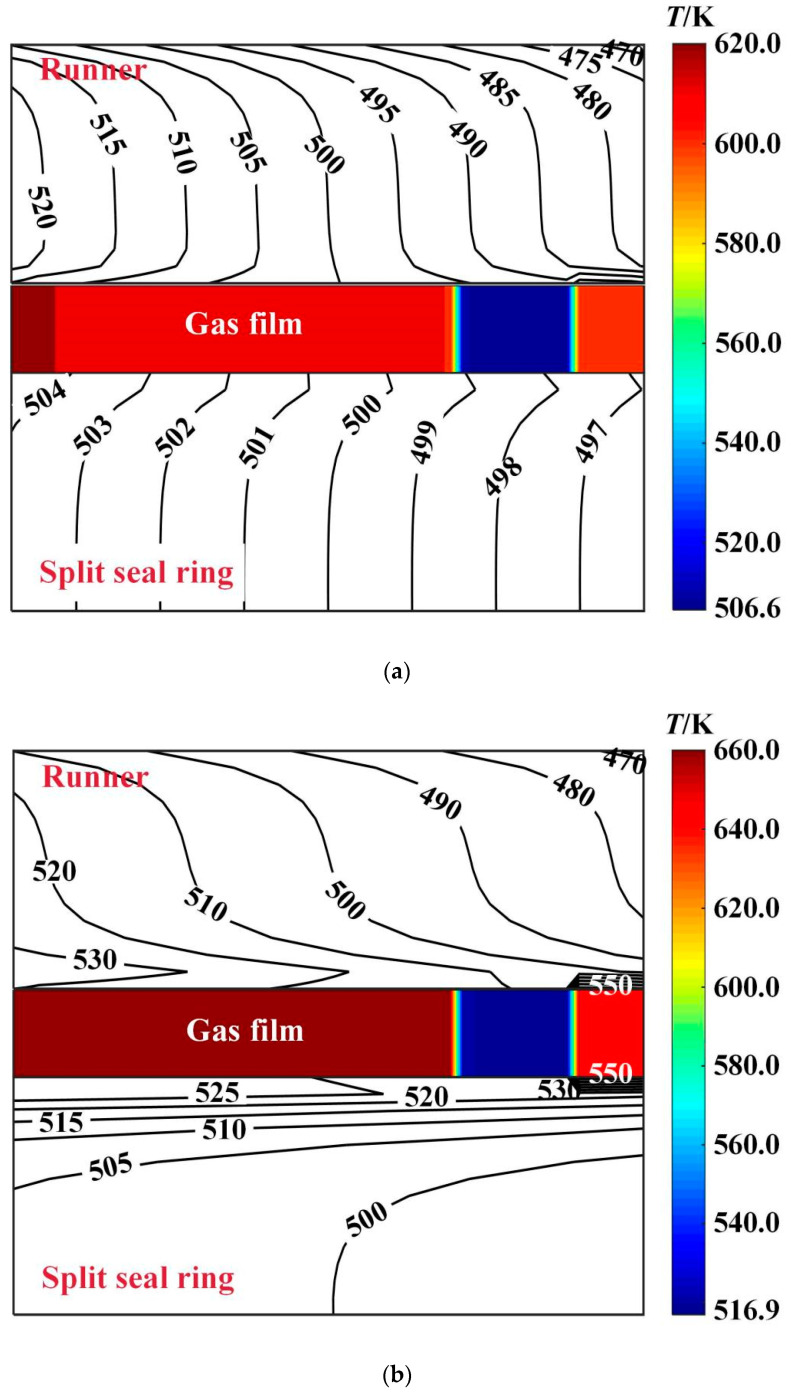
Influence of rotation speed on the maximum temperature of gas film. (*p*_i_ = 0.2 MPa, *ω* = 3.0 × 10^4^ r/min, *T*_in_ = 580 K, *T*_out_ = 420 K). (**a**) *β* = 6 × 10^−6^ rad; (**b**) *β* = 0.

Figure 17 shows the temperature distribution of the center profile of sealing friction pair at higher ambient temperature of 420 K. It is shown that, the ambient temperature has obvious influence on the temperature distribution of sealing friction pair. When the deflection angle changes from 0 to 6 × 10^−6^ rad, the maximum temperature of the gas film decreases from 660.0 K to 620.0 K, a decrease of about 6.1%. The runner temperature decreases from 550.0 K to 520.0 K, a decrease of about 5.5%, while the seal ring temperature is reduced from 550.0 K to 504.0 K, a decrease of about 8.4%.

**Figure 18:**
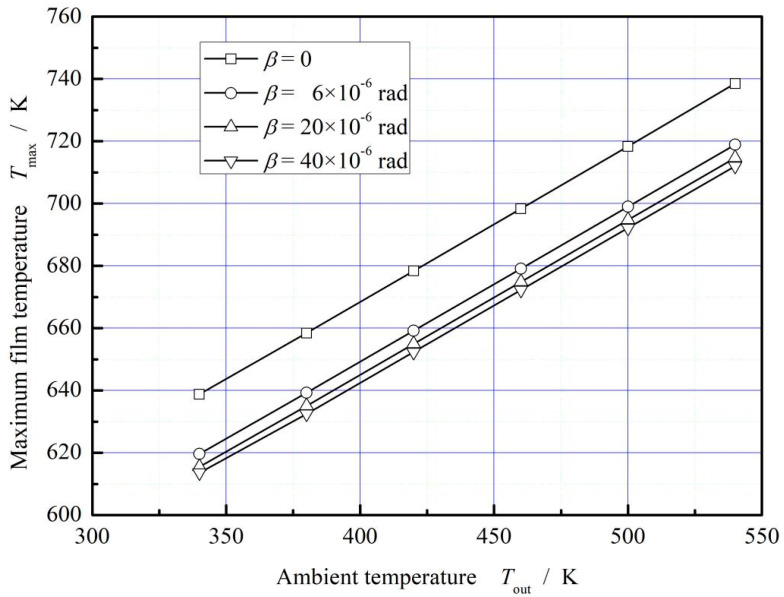
Influence of rotation speed on the maximum temperature of gas film. (*p*_i_ = 0.2 MPa, *ω* = 3.0 × 10^4^ r/min, *T*_in_ = 580 K).

Figure 18 plots the effect of ambient temperature on the maximum film temperature. The maximum film temperature increases obviously with the increase of ambient temperature. Compared with the absence of ring deflection, the increase of ring deflection angle has a significant decreasing trend on the maximum temperature of the film, and the maximum cooling range is about 3.6%.

**Figure 19:**
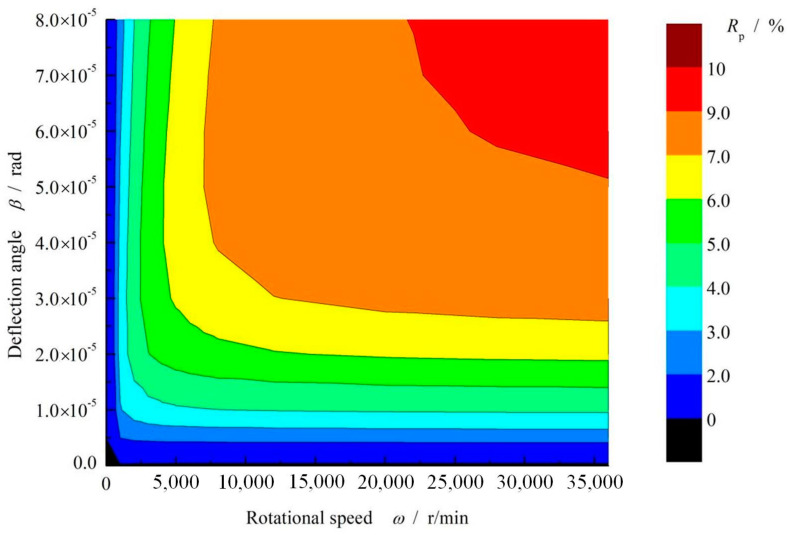
Selection interval diagram of dimensionless mean pressure increase rate. (*p*_i_ = 0.2 MPa, *T*_in_ = 580 K, *T*_out_ = 380 K, *p*_s_ = 0.03 MPa).

As discussed above, deflection angle and rotational speed are two most important parameters that affect thermo-hydrodynamic effect in the gas split ring seals. So, the increase rate of average pressure due to hydrodynamic effect is presented under different rotational speed and deflection angle, as shown in Figure 19. It is shown that the hydrodynamic effect increases with increasing deflection angle as well as rotational speed. In the high temperature case, an 10% increase rate of average pressure can be obtained with increase of both deflection angle and rotational speed.

## 4. Conclusions

(1) The surface Rayleigh step grooves do not present obvious hydrodynamic effect if split seal ring has no deflection. However, significant hydrodynamic effect can be obtained when the split seal ring presents a deflection angle of dozens of micro radians. Here, an increase 10% of the opening force is achieved when the deflection angle reaches 80 μrad in the case of speed 30,000 r/min and seal pressure 0.2 MPa.

(2) The hydrodynamic effect increases with increasing deflection angle as well as rotational speed. Meanwhile, the increase of rotational speed results in an obvious increase of film temperature. Besides, the increase of the ambient temperature leads to significant decrease of leakage rate, exceeding 50% with the ambient temperature increasing from 340 K to 540 K, but do not present obvious effect on the opening force.

## Figures and Tables

**Table 1:** Main parameters of the seal ring and Rayleigh step groove.

Item	Value
Inner radius of seal ring *r*_i/_mm	56
Thickness of seal ring *L*_/_mm	8.5
Number of split ring *N*	3
Groove number *n*	5
Rayleigh step groove width *R*_w/_mm	1.37
Circumferential groove width *C*_w/_mm	1.5
Groove depth *h*_d/_mm	0.8

**Table 2:** Characteristics of the materials [22].

Item	Graphite Ring	Runner
Density/kg·m^−3^	1800	7800
Young’s modulus/GPa	25	204
Poisson’s coefficient	0.20	0.3
Specific heat capacity/J·kg^−1^·K^−1^	710	460
Thermal conductivity/W·m^−1^·K^−1^	15	16.4
Linear thermal expansion coefficient/10^−6^ K^−1^	4	15.9

## Data Availability

All data is contained within the article.
